# Acoustic streaming enabled moderate swimming exercise reduces neurodegeneration in *C. elegans*

**DOI:** 10.1126/sciadv.adf5056

**Published:** 2023-02-22

**Authors:** Joyita Bhadra, Nakul Sridhar, Apresio Kefin Fajrial, Nia Hammond, Ding Xue, Xiaoyun Ding

**Affiliations:** ^1^ Department of Molecular, Cellular and Developmental Biology, University of Colorado, Boulder, CO 80309, USA.; ^2^ Department of Mechanical Engineering, University of Colorado, 1111 Engineering Dr., Boulder, CO 80309, USA.; ^3^ Biomedical Engineering Program, University of Colorado, Boulder, CO 80309, USA.; ^4^ BioFrontiers Institute, University of Colorado, Boulder, CO 80309, USA.

## Abstract

Regular physical exercise has been shown to delay and alleviate neurodegenerative diseases. Yet, optimum physical exercise conditions that provide neuronal protection and exercise-related factors remain poorly understood. Here, we create an Acoustic Gym on a chip through the surface acoustic wave (SAW) microfluidic technology to precisely control the duration and intensity of swimming exercise of model organisms. We find that precisely dosed swimming exercise enabled by acoustic streaming decreases neuronal loss in two different neurodegenerative disease models of *Caenorhabditis elegans*, a Parkinson’s disease model and a tauopathy model. These findings highlight the importance of optimum exercise conditions for effective neuronal protection, a key characteristic of healthy aging in the elderly population. This SAW device also paves avenues for screening for compounds that can enhance or replace the beneficial effects of exercise and for identifying drug targets for treating neurodegenerative diseases.

## Teaser

The Acoustic Gym technology reveals that precisely dosed moderate swimming exercise reduces neurodegeneration in *C. elegans*.

## INTRODUCTION

In recent years, physical exercise has been shown to be an accessible and effective form of therapy to treat many cardiovascular and metabolic diseases, type 2 diabetes, neurological diseases, cancer, and age-related decline in muscle, immune, and mental functions (*1*–*4*). Neurodegenerative diseases like Parkinson’s disease (PD) and Alzheimer’s disease (AD), prevalent in people over the age of 60, are characterized by progressive and permanent loss of neurons, for which there is currently no cure. Studies have shown that exercise can delay the onset of some neurological diseases while reducing side effects in others (*1*). Blood plasma from mice that had exercised has been shown to increase neurogenesis in aged recipient mice (*5*). Another study in mice showed that exercise plasma can reduce neuroinflammatory gene expression in the brain (*6*). Despite these potential benefits of exercise to alleviate disease progression and symptoms, many patients, particularly those who are older with severe symptoms, may not be able to follow certain workout regimens due to poor health and physical weakness. It will be important to identify exercise-related factors and mechanisms so that alternative therapeutic strategies can be developed to achieve similar outcomes.

*Caenorhabditis elegans* is an excellent animal model to study various neurodegenerative diseases such as PD, AD, and other forms of tauopathies (*7*–*9*). The age-related decline of multiple tissues and bodily functions in *C. elegans* has been well studied (*10*, *11*). It has been reported that *C. elegans* can gain beneficial effects of exercise through swimming in liquid (*12*, *13*). The current methods of exercise for *C. elegans*, however, rely mainly on long-term passive swimming for several hours per day. Not only is the process relatively labor-intensive and time-consuming, but also such passive swimming exercise cannot control the exercise intensity. To determine specific exercise dosages that are most beneficial to animal health, a tool to stimulate consistent and uniform swimming exercise with controllable workout is crucial.

Microfluidic devices have long been used to immobilize worms (*14*–*16*), sort and screen worms (*17*, *18*), and study worm behavioral responses to controlled stimuli (*19*, *20*). Integrating surface acoustic wave (SAW) technology into a microfluidic platform has enabled on-chip manipulation of fluids, nano/microparticles, cells, and small animals (*21*–*24*). Technical benefits of using SAW in microfluidics include low toxicity, low cost, miniaturized setup, and precise control over the acoustic field for particle and fluid actuation (*21*). Moreover, SAW microfluidics has been previously used for spatial and rotational manipulation of *C. elegans* animals, and the system itself demonstrates high biocompatibility (*25*, *26*).

Here, we create the Acoustic Gym, a SAW-integrated microfluidics system that can actively stimulate swimming exercise of a large number of worms at desired intensities and durations. We demonstrate that this Acoustic Gym can identify the optimal conditions for improving neuronal health in two different models of neurodegeneration in *C. elegans*, which unexpectedly require the same optimal conditions to achieve beneficial outcomes. Our results highlight the importance of optimum exercise conditions for neuronal protection and the potential utility of the Acoustic Gym in screening for compounds that can enhance or replace the beneficial effects of exercise, especially in the older population where exercise is not always a practical solution, and for identification of drug targets in treating neurodegenerative diseases.

## RESULTS

### Acoustic gym design and working principle

**Fig. 1.**
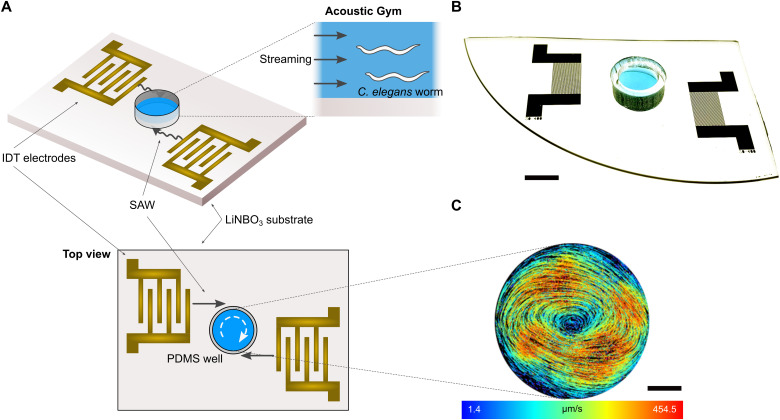
Schematic and working principle of Acoustic Gym microfluidic device that applies SAW with controlled intensity and duration. (**A** and **B**) Schematic (A) and photo (B) of SAW device illustrating offset IDTs that produce clockwise streaming in the PDMS chamber. Scale bar, 6 mm. (**C**) Top view velocity colormap produced by tracking microparticles (*n* = 5264) dispersed in fluid under 0.125-W, 50% duty cycle showing an even distribution of streaming throughout the chamber area. Scale bar, 1 mm.

The design and working mechanism of the Acoustic Gym are shown in Fig. 1 (A and B). The SAW microfluidic device consists of a circular polydimethylsiloxane (PDMS) chamber filled with fluid, which is placed in the center between a pair of offset interdigital transducers (IDTs) that are deposited on a lithium niobate (LiNbO_3_) substrate (Fig. 1A). Upon actuation (18 MHz) from a radio frequency (RF) source, the SAW propagates along the substrate toward the PDMS chamber (Fig. 1, A and B). Once the SAW reaches the chamber, the difference in velocity of acoustic propagation along the fluid versus the substrate causes leakage of some of the wave energy into the fluid. This induces a clockwise rotational acoustic streaming in the fluid inside the chamber (Fig. 1A and movie S1A). The streaming velocity can be precisely controlled via the RF signal input power.

### Characterization of acoustic streaming and temperature increase in SAW chamber

**Fig. 2.**
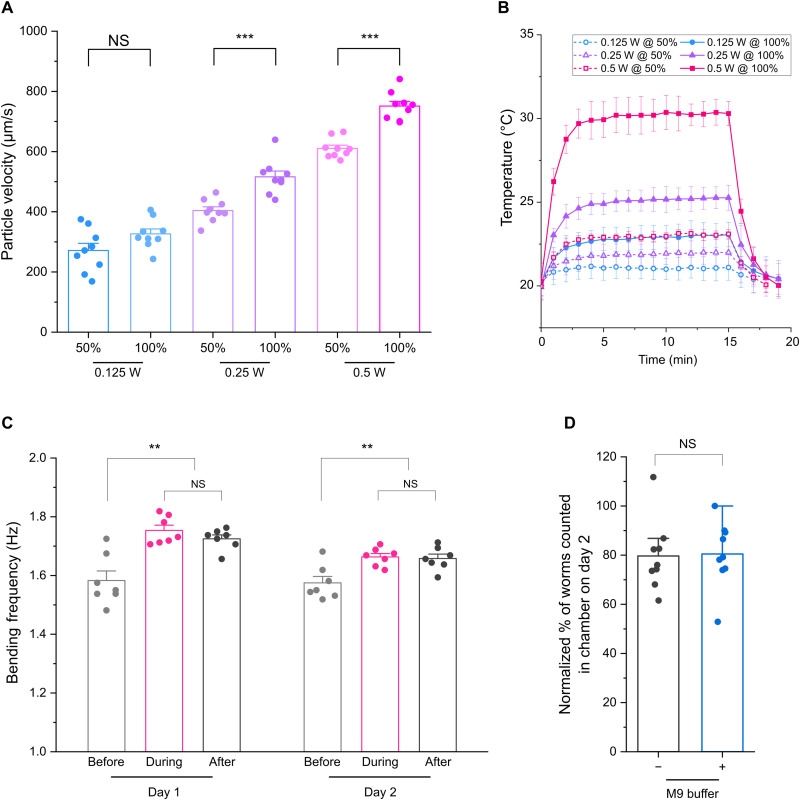
Characterization of Acoustic Gym. (**A**) Mean instantaneous particle velocity for different RF input powers and duty cycles, allowing for precise controllability of streaming in the device. (**B**) Thermal characterization for different RF input powers and duty cycles (15 min in each). (**C**) Bending frequency of animals swimming in the SAW chamber taken immediately before SAW was applied, after 2 min of SAW application, and 2 min after SAW was turned off in a 2-day, 5-min-per-day schedule (see Materials and Methods). (**D**) Percentage of animal survival in 60 μl of M9 media with (+) or without (−) the SAW treatment in adult day 2, compared with that of adult day 1 set at 100%. SAW treatment was at 0.125-W, 50% duty cycle for 5 min. Data presented are means ± SEM. For (A) and (D), at least nine independent experiments were performed in each condition or time point. For (B), three independent measurements of temperature profile were taken. For (C), at least seven independent experiments were performed in each condition or time point. **P < 0.01 and ***P < 0.001; two-sided, unpaired *t* test. NS, not significant.

To test the controllability and to quantify the velocity distribution across the area of the SAW chamber, we dispersed 15-μm polystyrene microparticles in the M9 buffer to track the streaming velocity for various SAW power levels at both 50% and continuous (100%) RF input duty cycles (movie S1A). After acquiring videos of the streaming at different power levels, we analyzed the paths of individual particles to generate a velocity colormap (Fig. 1C). The velocity of each individual particle track was determined, and the mean velocity was calculated at each power level (Fig. 2A). Since acoustic waves propagating in a fluid medium disperse energy into the fluid as heat (*27*), we quantified the acoustic heating effects by applying SAW for 15 min at different power levels with two different duty cycles and tracking the temperature increase over time, as well as their cool-down after the SAW was turned off (Fig. 2B). Because *C. elegans* are sensitive to temperature increases (*28*, *29*), it is important to keep the heating effect minimal for accurate analysis.

The results showed that reducing the duty cycle from 100 to 50% at the same power level slightly decreased the streaming velocity, by 17 to 22% (Fig. 2A), but substantially decreased heating effects by 64 to 71% (Fig. 2B). The velocity tracking showed that the streaming velocity is stable across most of the chamber area, with the exception of a small region in the center and the edges near the outer wall (Fig. 1C). This representative example of the streaming pattern, using the 0.125-W, 50% duty cycle power level, showed a mean velocity of 284 μm/s and an SD of 92 μm/s after tracking approximately 5000 individual particles. These results indicate that the animals in the chamber would receive the similar amount of exposure to the SAW energy over time on average. We decided to use the 0.125-W, 50% duty cycle power level for most of the worm swimming exercise experiments since it represents a good balance between strong acoustic streaming and negligible temperature increase (Fig. 2B).

To assess the device’s ability to propagate acoustic energy in the natural growth environment of *C. elegans*, we replaced the M9 buffer in the chamber with 1% agarose gel and compared particle velocity and temperature increase against those in the fluid at 0.125-W, 50% duty cycle (movie S1, A and B). The results showed no particle streaming unless a layer of fluid was added on top of the gel medium (movie S1B), indicating that SAW works best with the liquid medium. Once fluid was added (movie S1C), particle velocity and temperature change showed no significant difference compared to those in the fluid in the chamber without gel (fig. S1, A and B), indicating that acoustic energy losses through the gel were minimal.

### SAW treatment increases animal activity and preserves their viability

Using the 0.125-W, 50% duty cycle power level of SAW, we tracked the bending frequency of both L4 and adult day 1 animals, which reflects the exercise intensity, in the Acoustic Gym before (movie S2A), during (movie S2B), and after SAW applications (movie S2C) over a 5-min-per-day, 2-day exercise schedule. The results showed a significant increase in bending frequency during and after SAW applications (Fig. 2C), when compared to before SAW applications, suggesting that streaming in the chamber with SAW provides a stimulus for more intense exercise compared with animals allowed to swim freely in the chamber.

We next investigated the effect of SAW on the viability of animals by quantifying live animals in the chamber on days 1 and 2 to determine the survival rate of animals with and without SAW treatment (Fig. 2D). There was no viability difference between SAW and no SAW treatments, suggesting that the SAW treatment did not affect animal viability. It is noteworthy to mention that in our SAW experiments in liquid, there is a consistent loss of approximately 20% of animals between days 1 and 2, due to adherence of animals to pipette tips (*30*), nematode growth medium (NGM) plates, and/or the SAW chamber (Fig. 2D).

### Proper duration and intensity of SAW are crucial for neuronal protection

**Fig. 3.**
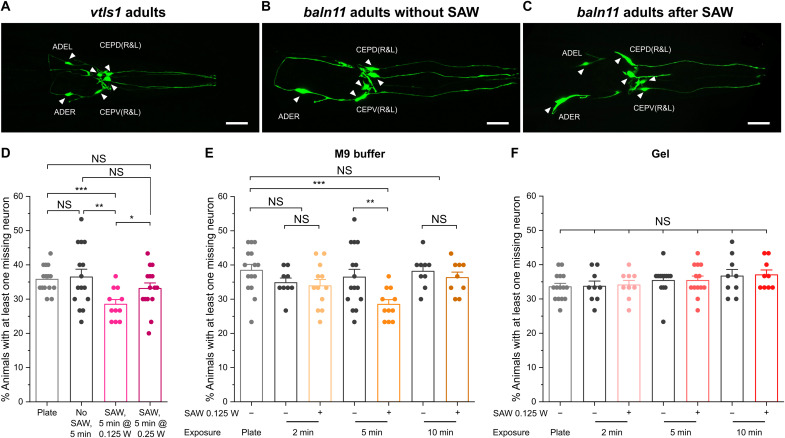
Characterization of dopaminergic neuronal loss following exercise using Acoustic Gym. (**A**) A representative image of the six head DA neurons in *vtIs1* (P*dat-1*::GFP) adult day 2 animals. (**B** and **C**) Representative images of DA neurons in adult day 2 *baIn11* (P*dat-1*::α-syn/P*dat-1*::GFP) animals, without (B) and with SAW treatment at 0.125-W, 50% duty cycle for 5 min (C). The Anterior Deirid Neuron Left (ADEL) was lost in (B) and no DA neuron loss in (C). The six neurons depicted in (A-C) are Anterior Deirid Neuron Right (ADER), ADEL, Cephalic Sensory Neuron Ventral Right & Left [CEPV(R&L)], and Cephalic Sensory Neuron Dorsal Right & Left [CEPD(R&L)]. Scale bars, 20 μm. (**D**) Comparison of DA neuronal loss in adult day 2 *baIn11* animals grown on the NGM plate (Plate) or in the SAW chamber in M9 buffer without or with SAW treatment at two different intensities as indicated. (**E**) Comparison of DA neuronal loss in adult day 2 *baIn11* animals grown on the NGM plate (Plate) or in the SAW chamber in M9 buffer without (−) or with (+) SAW treatment in three different durations as indicated. (**F**) Comparison of DA neuronal loss in adult day 2 *baIn11* animals grown on the NGM plate (Plate) or in the SAW chamber on the gel without (−) or with (+) SAW in three different durations as indicated. Data are means ± SEM. At least nine independent experiments were performed in each stage or condition. For each independent experiment, at least 30 animals were scored. *P < 0.05, **P < 0.01, and ***P < 0.001; two-sided, unpaired *t* test.

*C. elegans* has eight dopaminergic (DA) neurons, six in the head—four cephalic sensory neurons and two anterior deirid neurons—and two in the mid-section—posterior deirid neurons. For this study, we focused on the six head DA neurons, which can be labeled by an integrated transgene *vtIs1* containing the P*dat-1*::GFP reporter (Fig. 3A), a transcriptional green fluorescent protein (GFP) fusion of the *dat-1* gene that encodes a dopamine transporter and is expressed only in eight DA neurons (*31*). We used a widely used *C. elegans* PD model (Fig. 3, B and C), the *baIn11* strain (P*dat-1::*α*-syn*/P*dat-1::*GFP) (*7*), in which human α-synuclein (α-syn), an aggregation-prone protein associated with PD (*32*), and GFP are coexpressed under the control of the *dat-1* gene promoter through an integrated transgene. Previous reports show that *baIn11* animals undergo significant DA neuronal loss (*33*, *34*). For example, approximately 36% of adult day 2 *baIn11* animals grown on NGM plates have at least one missing DA neuron (Fig. 3D).

To investigate the neuroprotective effect of exercise, we tested a variety of SAW durations and intensities on *baIn11* animals in the Acoustic Gym. When *baIn11* animals were subjected to a 5-min exercise each day induced by SAW at 0.125-W, 50% duty cycle power for 2 days, significant reduction of DA neuronal loss (28%) occurred (Fig. 3D). In comparison, no reduction in DA neuronal loss was observed when animals were allowed to swim freely in the SAW chamber without the application of SAW in the same 5-min, 2-day schedule, indicating that exercise with a consistent intensity may be required to improve DA neuronal survival. SAW treatment for shorter (2 min) or longer (10 min) duration did not improve DA neuronal survival compared with their corresponding no SAW controls (Fig. 3E). In addition, increasing the workout intensity by raising the SAW power level to 0.25 W with the same 50% duty cycle for 5 min, which increased the fluid streaming speed (Fig. 2A), did not consistently reduce DA neuronal loss (Fig. 3D). These findings suggest that a proper regime of workout duration and intensity could be important to effectively reduce DA neuronal loss in *baIn11* animals.

When the liquid medium in the SAW chamber was replaced with 1% agarose gel, no rescue of DA neuron loss was seen with the same 2-day SAW treatment at 0.125-W, 50% duty cycle for 5 min each day or with a shorter (2 min) or longer (10 min) SAW treatment (Fig. 3F). In this gel-based experiment, SAW propagated through the gel without inducing streaming (movie S1B), and animals were exposed to the acoustic field without being forced to swim. Particle tracking comparisons showed that SAW was able to propagate through the gel and produce similar acoustic energy on the animals when compared with the liquid medium (movie S1C). On the basis of these results, the main factor responsible for improving neuronal protection is the exercise, not the presence of SAW. Under Acoustic Gym exercise, we observe that animals are swimming against the fluid stream direction (movie S2B). This positive rheotaxis has been reported previously in which *C. elegans* animals reorient their bodies toward the direction of fluid flow current (*35*).

### SAW-induced exercise improves neuronal health in a *C. elegans* tauopathy model

**Fig. 4.**
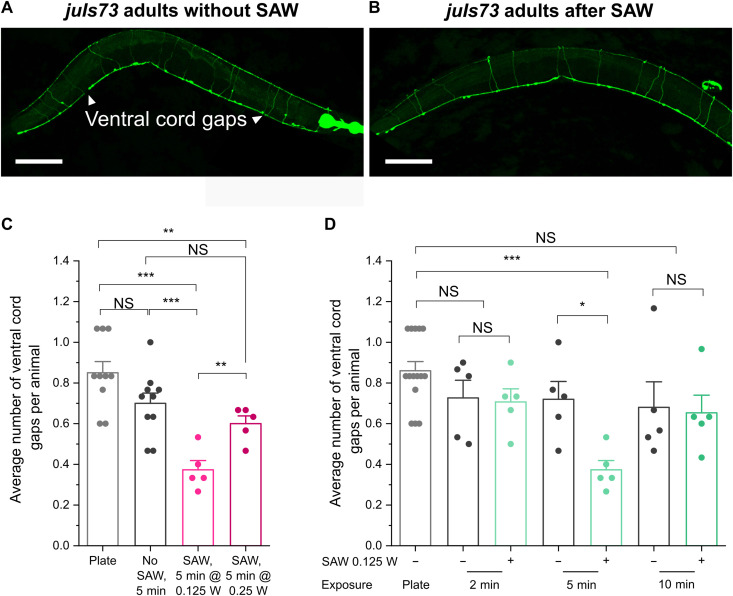
Characterization of neuronal health in animals expressing pro-aggregating tau fragments following exercise in Acoustic Gym. (**A** and **B**) Representative images of adult day 3 animals with pan-neuronal expression of aggregating tau and GFP expression in GABAergic motor neurons without and with SAW treatment as indicated. Arrowheads indicate gaps in ventral nerve cords, which were frequently observed in animal with no SAW treatment (A) but less in the animal treated with SAW for 5 min at 0.125-W and 50% duty cycle (B). Scale bars, 100 μm. (**C**) Comparison of the average number of ventral nerve cord gaps in adult day 3 animals grown on the NGM plate (Plate) or placed in the SAW chamber in M9 buffer without or with SAW treatment at two different intensities as indicated. (**D**) Comparison of the average number of ventral nerve cord gaps in adult day 3 animals grown on the NGM plate (Plate) or in the SAW chamber in M9 buffer without (−) or with (+) SAW treatment in three different durations as indicated. Data are means ± SEM. At least five independent experiments were performed in each stage or condition. For each independent experiment, at least 30 animals were scored. *P < 0.05, **P < 0.01, and ***P < 0.001; two-sided, unpaired *t* test.

We further validated the neuroprotective effect of active swimming exercise in the Acoustic Gym on a tauopathy model of *C. elegans*. Hyperphosphorylated, insoluble, and filamentous tau is a hallmark of many neurodegenerative disorders, collectively known as tauopathies (*36*). These disorders include, but are not limited to, AD, amyotrophic lateral sclerosis, certain prion diseases, and some PD (*36*–*39*). For our experiments, we selected a tauopathy model in which human pro-aggregating tau fragment, F3ΔK280, and the full-length mutant tau protein (V337M) were coexpressed in all neurons, and the GABAergic (γ-aminobutyric acid–releasing) motor neurons are labeled by GFP (P*rab-3::F3*Δ*K280*; P*aex-3::h4R1NTauV337M*; P*unc-25::GFP*) (Fig. 4, A and B) (*9*). This strain shows severe neuronal defects, such as gaps in nerve cords during larval and adult stages (Fig. 4A). Similar to what we found with the PD model, we observed a significant decrease in ventral nerve cord gaps in adult day 3 animals with the exercise program of SAW at 0.125-W and 50% duty cycle for 5 min each day for 2 days (Fig. 4, B to D). Higher intensity of SAW at 0.25 W for the same duration was less effective in reducing the ventral nerve cord gaps but still showed significant improvement when compared with animals grown on the NGM plates or with no SAW treatment (Fig. 4C). Similar to what we have observed in the PD model, shorter (2 min) and longer (10 min) SAW treatment at the same 0.125-W intensity failed to reduce ventral cord gaps (Fig. 4D), again indicating that proper exercise duration is crucial for inducing a beneficial effect. Together, our findings in two different neurodegeneration models indicate that moderating the intensity and duration of exercise is vital for optimizing its neuronal health benefits, and such benefits may be regulated by a common pathway or factors, as the same exercise intensity and duration are required to achieve optimal neuronal protection in two very different models of neurodegenerative disease.

## DISCUSSION

It is generally accepted that exercise can improve human and animal health (*2*–*4*, *40*, *41*). Numerous reports suggest that moderate exercise induces neuroplasticity and neuronal repair, improves memories, and bestows other beneficial effects in humans and in other animal models (*1*, *5*, *6*). Exercise has also been shown to alleviate neurodegenerative diseases (*42*–*44*). For example, DA neurons are more susceptible to the impact of exercise, leading to the mitigation of PD symptoms (*42*, *43*). In *C. elegans*, passive swimming for 15 to 20 min has been shown to reduce the α-syn protein level (*44*). Similarly, exercise has been shown to reduce tau hyperphosphorylation, attenuating the progression of tauopathies (*45*). Nevertheless, how exercise can improve human health, including neuronal health, remains poorly understood. Moreover, it has been reported that exercise intensity and duration could directly affect the outcomes of exercise and even have adverse effects (*46*–*48*). Therefore, identification of the proper workout condition is vitally important.

To answer this question and reveal the right workout condition that is most beneficial, a tool to precisely control the exercise regimen is necessary. The Acoustic Gym provides such an ideal experimental system. The Acoustic Gym can generate consistent and uniform swimming exercise for large groups of animals and allows precise control of both the duration and intensity of swimming. Unlike passive swimming exercise (*12*, *13*), the Acoustic Gym exposes the animals to a continuous external stimulus, forcing them to exercise for the whole workout period.

To test whether controlled exercise provides neuronal protection in *C. elegans*, we used two different *C. elegans* neurodegeneration models, the PD model induced by human α-syn and a *C. elegans* tauopathy model. In the PD model, swimming exercise at the right intensity and duration can significantly reduce DA neuronal loss, compared with those seen in animals swimming freely in the liquid medium or crawling on the solid gel medium. In the tauopathy model, swimming exercise at the right intensity and duration again significantly reduces ventral nerve cord gaps, compared with those observed in passively swimming animals or animals grown on NGM plates. Because neurodegeneration in these two models is caused by different factors and mechanisms, controlled exercise must affect factors or pathways that are crucial for protection against both neurodegeneration events. The Acoustic Gym offers an excellent platform to facilitate identification of such factors or pathways using the powerful genetic and biochemical approaches in *C. elegans*.

In both neurodegenerative disease models, the same optimum exercise intensity and duration (0.125 W for 5 min each day for 2 days) are required for significant neuronal protection. Slightly shorter (2 min) or longer (10 min) exercise or more intense workout (0.25 W) did not protect or failed to produce optimal neuronal protection. These findings suggest that neurons prone to degeneration in these two distinct disease models are sensitive to subtle changes of exercise regimen and the corresponding molecular changes, which could affect factors that are both pro- and antineuronal health, and the collective influences of these factors determine whether a beneficial outcome is achieved. For example, longer or more intense exercise could increase oxygen consumption and the production of reactive oxygen species (*46*–*48*), which promote oxidative damage (*49*) and antagonize the beneficial outcome of pro-survival factors. The intricate nature of exercise partially explains why there is a huge variation in beneficial effects of exercise in data accumulated from humans (*50*). It also makes *C. elegans* and the Acoustic Gym the unparalleled animal model and technology to study the complex mechanisms of exercise-based health improvement and negative effects stemming from too much exercise. Future improvements of the device could integrate a cooling system to dissipate excess SAW-induced heat (*51*, *52**)*, thereby allowing for faster streaming with higher SAW inputs and examination of the mechanistic basis of associated negative effects.

The prevalence and severity of neurodegenerative diseases are higher in older population. Meanwhile, for the elderly people, the ability and willingness to regularly exercise decrease due to physical frailty, especially in patients with PD (*53*). Forced exercise has been shown to reduce PD-related motor symptoms (*54*, *55*). But this kind of forced exercise regimens for older individuals have to be supervised by trained professionals, which is not always possible. It is already challenging to find a single optimum workout condition even for young and healthy humans. For patients, the type of workout and their physical ability must be considered while devising an optimal exercise regimen to delay the onset of neurodegeneration. Moreover, what is ideal for one person might be detrimental for others. This complicated relationship and conundrum between the disease and the treatment can be resolved by identifying specific beneficial and adverse factors induced by exercise so that therapeutic solutions can be developed to maximize the impact of the beneficial factors and to minimize the production and influence of the adverse factors. Again, *C. elegans* and the Acoustic Gym will be the ideal platform to identify these genes through genetic screens, RNA interference screens, RNA sequencing analysis, and proteomic analysis. This unique platform can also facilitate high-throughput drug screens to identify compounds that enhance the beneficial effects of light exercise or that can even replace the need of exercise if physical conditions are not permissive (*56*, *57*).

## MATERIALS AND METHODS

### Device fabrication

The device consists of two major parts: the lithium niobate substrate patterned with the offset pair of IDTs and the PDMS chamber. The LiNbO_3_ substrate was patterned using standard photolithography techniques. First, a layer of photoresist (S1813, Dow, USA) was spin-coated onto a 76.2 mm, Y+36° X-propagation LiNbO_3_ wafer. The IDT structures were then patterned onto the wafer using ultraviolet light exposure and development in a photoresist developer (MF319, Dow, USA). Next, layers of chrome and gold (Cr/Au, 10/100 nm) were deposited using e-beam evaporation. Excess photoresist was removed with a lift-off process (Remover PG, Kayaku, Japan). Last, the wafer was diced into multiple individual devices each with a pair of offset IDTs. Each individual IDT consists of 20 electrode pairs with a spacing of 50 μm and an aperture of 6 mm. The offset distance between each pair of IDTs is 3 mm, and the horizontal distance is 18 mm.

The PDMS chamber was fabricated by pouring a mixture of PDMS base and cross-linker (Sylgard 184, Dow Corning, USA) with a ratio of 10:1 (w/w) into a standard plastic petri dish. The height of the chamber was approximately 3 mm. The mixture was then placed under vacuum for 30 min to remove air bubbles and then cured at 65°C for 1 hour. An 8-mm biopsy hole puncher was used to punch out the outer diameter of chamber, while a 6-mm puncher was used for the inner diameter. The chamber was then bonded to the prepared LiNbO_3_ substrate after treatment with oxygen plasma (PDC-001, Harrick Plasma, USA). To ensure strong bonding, the device was baked overnight at 65°C.

### Device operation

An RF function generator (N5171B, Keysight, USA) and power amplifier (403LA, E&I, USA) were used to input the signal into the IDTs. To identify the resonant frequency of the devices, a network analyzer (E5061B, Keysight, USA) was used in combination with visually tracking the fastest velocity of streaming microparticles. Depending on the individual device, the resonant frequency was identified as approximately 18.2 MHz.

All SAW experiments were conducted on an inverted microscope (Eclipse Ti2, Nikon, Japan). The chamber was imaged with a digital complementary metal-oxide semiconductor camera (Orca-Flash 4.0LT, Hamamatsu, Japan) in combination with the included imaging software (HCImage Live, Hamamatsu, Japan). Example videos with worms were recorded at 10 frames s^−1^. Videos for particle characterization were recorded at 33 frames s^−1^. Further processing of images and videos was done with ImageJ (National Institutes of Health, USA).

### Device characterization

Polystyrene microparticles (15 μm; Polysciences, USA) were diluted (1:10, v/v) and dispersed into 60 μl of M9 buffer (22 mM KH_2_PO_4_, 42 mM Na_2_HPO_4_, 8.6 mM NaCl, and 18.7 mM NH_4_Cl) to characterize the fluid streaming in the chamber. ImageJ TrackMate software was used to analyze the path of approximately 5000 particles to generate the velocity colormap (*58*). The instantaneous velocity of each individual particle track was then averaged together to calculate the overall mean velocity for each power level. To monitor the chamber temperature while applying SAW and during cool down, a digital thermal probe was used.

### Strains and maintenance

*C. elegans* strains were maintained at 20°C using standard methods (*59*). We used the N2 Bristol strain as the wild-type strain. The following alleles were used in this study: *vtIs1*[P*dat-1*::GFP + *rol-6(su1006)*] V, UA44 (*baIn11*[P*dat-1::*α*-syn* + P*dat-1::*GFP]), and BR5707 (*byIs161*[P*rab-3::F3*Δ*K280;* P*myo-2::mCherry*]; *bkIs10*[P*aex-3::h4R1NTauV337M;* P*myo-2::GFP*]; *juIs73*[P*unc-25::GFP*] III).

### Quantification of DA neuron survival

The UA44 strain, in which GFP and α-syn are coexpressed in *C. elegans* DA neurons (*baIn11*) (*7*), and the *vtIs1* strain, where only the GFP is expressed in the DA neurons, were synchronized by bleaching with 20% alkaline hypochlorite solution (3 ml of bleach + 3.75 ml of 1 M NaOH + 8.25 ml H_2_O). The resulting L1 larvae were transferred to OP50 (an *Escherichia coli* strain) bacteria–seeded NGM plates. The animals were scored for DA neuronal loss at adult day 2 with or without SAW treatment using 40× optic of the Zeiss Axioplan 2 microscope equipped with epifluorescence.

### Quantification of ventral cord gaps

In BR5707 strain, because of tau aggregation, ventral and dorsal cord gaps can be observed during larval and adult stages using a GFP reporter (*9*). We used the same bleaching and plating technique as UA44. A number of ventral cord gaps were scored on adult day 3 with or without SAW treatment using 40× optic of the Zeiss Axioplan 2 microscope equipped with epifluorescence.

### Quantification of viability of animals after SAW treatment

Images of the SAW chamber containing animals were taken before SAW treatment on both days 1 and 2, in M9 buffer. For each chamber image, worms were manually counted from three randomly selected regions of interest (0.5 by 0.5 mm). The raw animal counts were normalized to the average day 1 values to calculate a percent loss of animals between days 1 and 2.

### Quantification of the bending frequency of animals

Synchronized L4 stage N2 animals were washed off the NGM plates, and approximately, 30 animals were transfer to the SAW chamber in 60 μl of M9 buffer. Bright-field videos of animals swimming in the chamber were taken immediately before SAW was turned on, during SAW on (2.5 min), and 1 min after SAW was turned off. All videos were captured for 15 s at 10 frames s^−1^ using the 2× optic of the inverted Nikon Eclipse Ti2 microscope. Average bending frequency was calculated by manually tracking the total dorsal-ventral bends in individual animals and dividing by the total time frame (15 s). The same procedure was repeated the next day, at adult day 1.

### Treatment with SAW in fluid

Synchronized animals were allowed to grow until L4 larval stage (UA44) or adult day 1 stage (BR5707) in normal NGM plates. They were then washed off the NGM plates in M9 buffer, and approximately, 500 animals were transferred to the SAW chamber in 60 μl of M9 buffer along with the OP50 bacteria from the wash to avoid starvation. SAW at different intensity and duration was applied. The animals were then transferred back to freshly seeded NGM plates. The duration that the animals spent in the SAW chamber was short (around 10 min); thus, they had access to ample food during the whole time of exercise. The same procedure was repeated the next day, at adult day 1 (UA44) or adult day 2 (BR5707). DA neuronal loss for UA44 was scored on adult day 2. Ventral chord gaps in BR5707 were scored on adult day 3.

### Treatment with SAW on gel for UA44

Fifty microliters of 1% agarose was added to the SAW chamber and allowed to solidify. Synchronized L4 stage animals were washed off the NGM plates, and approximately, 100 animals were added in 10 μl of M9 buffer along with OP50 to the gel bed, and excess fluid was removed using Kimwipe. SAW at different intensity and duration was applied. Animals were then washed from the SAW chamber with the M9 buffer and transferred to fresh NGM plates. Using a tweezer, the gel in the SAW chamber was also transferred to the NGM plates to minimize animal loss. The same protocol was applied in the next day, on adult day 1. DA neuronal loss was scored on adult day 2.
