# Supplementary material for: Acoustic streaming enabled moderate swimming exercise reduces neurodegeneration in C. elegans
Source: Sci Adv. 2023 Feb 22;9(8):eadf5056. doi: 10.1126/sciadv.adf5056 (PMC9946341; doi:10.1126/sciadv.adf5056)
Supplement: Supplementary file 1 — Fig. S1 Legends for movies S1 and S2 [file sciadv.adf5056_sm.pdf]

Science Advances
AAAS

Supplementary Materials for

Acoustic streaming enabled moderate swimming exercise reduces neurodegeneration in *C. elegans*

Joyita Bhadra *et al.*

Corresponding author: Ding Xue, ding.xue@colorado.edu; Xiaoyun Ding, xiaoyun.ding@colorado.edu

Sci. Adv. **9**, eadf5056 (2023)
DOI: 10.1126/sciadv.adf5056

The PDF file includes:

Fig. S1
Legends for movies S1 and S2

Other Supplementary Material for this manuscript includes the following:

Movies S1 and S2

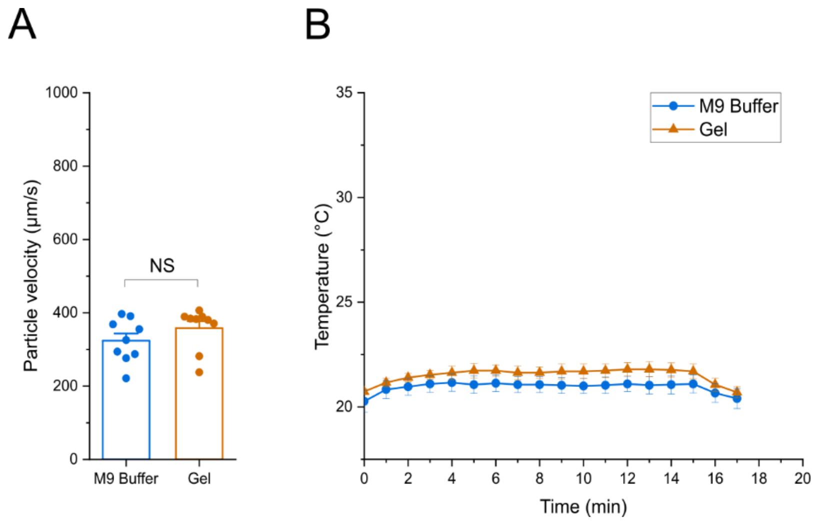

Fig. S1. Device performance comparison between M9 buffer and gel medium. Comparison of particle velocity (**A**), and temperature (**B**), in M9 buffer and gel under the 0.125W, 50% duty cycle SAW treatment. All experiments were done either in 60 µl of M9 media in chamber or 60 µl of M9 media on top of 50 µl of 1% agarose gel in chamber. For **A,** at least nine independent experiments were performed in each condition. For **B**, SAW was applied for 15 minutes, and three independent measurements of the temperature profiles were done. In all panels**,** data presented are mean ± s.e.m. NS, no significant difference, based on two-sided, unpaired *t*-test.

Movie S1 (separate file). Representative videos of particle movement during SAW treatment in different medium. Videos show how particles moved in the microfluidic device following SAW in liquid (60 µl of M9) **(A),** on the surface of solid (50 µl of 1% agarose gel) **(B),** and in liquid on the surface of solid (60 µl of M9 media on top of 50 µl of 1% agarose gel) **(C)**.

Movie S2 (separate file). Representative videos of worm movement during SAW applications. Videos show worm movement 2 minutes after their transfer to the SAW chamber **(A)**, 2 minutes after SAW was turned on **(B),** and 2 minutes after SAW was turned off **(C)**. SAW was applied at 0.125W, 50% duty cycle for 5 minutes in the M9 buffer.
